# 5-Cyano-1,3-phenyl­ene di­acetate

**DOI:** 10.1107/S1600536814011374

**Published:** 2014-05-24

**Authors:** Bahar Abbassi, Michela Brumfield, Lloyd M. Jones, Vladimir N. Nesterov, Andrew J. Carr

**Affiliations:** a Department of Chemistry, Austin College, 900 North Grand, Sherman, TX 75090-4400, USA; b Department of Chemistry, University of North Texas, 1155 Union Circle, #305070, Denton, TX 76203-5070, USA

## Abstract

In the title mol­ecule, C_11_H_9_NO_4_, the two acet­oxy groups are twisted from the plane of the benzene ring by 67.89 (4) and 53.30 (5)°. Both carbonyl groups are on the same side of the aromatic ring. In the crystal, weak C—H⋯O hydrogen bonds link mol­ecules into layers parallel to the *ac* plane. The crystal packing exhibits π–π inter­actions between the aromatic rings, indicated by a short inter­centroid distance of 3.767 (3) Å.

## Related literature   

For background to thermoreversible organogelator compounds, see: Carr (2008 ▶). For background to the synthesis, see: Ellis *et al.* (1976 ▶). For a review of the dehydration of amides to nitriles, see: Bhattacharyya *et al.* (2012 ▶). For the crystal structure of a related compound, see: Haines & Hughes (2009 ▶).
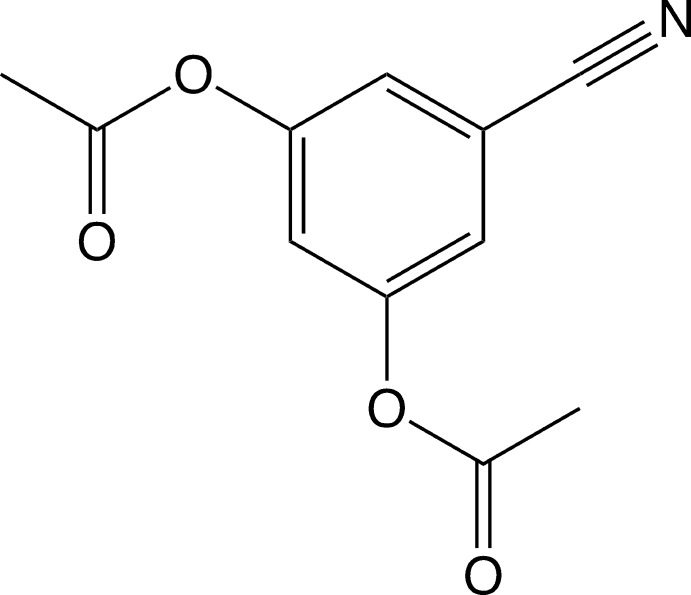


## Experimental   

### 

#### Crystal data  

C_11_H_9_NO_4_
*M* *_r_* = 219.19
Monoclinic, 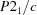

*a* = 6.2293 (5) Å
*b* = 21.1153 (17) Å
*c* = 8.5989 (7) Å
β = 109.171 (1)°
*V* = 1068.32 (15) Å^3^
*Z* = 4
Mo *K*α radiation
μ = 0.11 mm^−1^
*T* = 200 K
0.22 × 0.16 × 0.10 mm

#### Data collection  

Bruker APEXII CCD diffractometer
Absorption correction: multi-scan (*SADABS*; Bruker, 2007 ▶) *T*_min_ = 0.977, *T*_max_ = 0.990
14252 measured reflections
2340 independent reflections
2067 reflections with *I* > 2σ(*I*)
*R*_int_ = 0.025

#### Refinement  

*R*[*F* ^2^ > 2σ(*F* ^2^)] = 0.031
*wR*(*F* ^2^) = 0.092
*S* = 1.01
2340 reflections
148 parameters
H-atom parameters constrained
Δρ_max_ = 0.17 e Å^−3^
Δρ_min_ = −0.13 e Å^−3^

### 

Data collection: *APEX2* (Bruker, 2007 ▶); cell refinement: *SAINT* (Bruker, 2007 ▶); data reduction: *SAINT*; program(s) used to solve structure: *SHELXS97* (Sheldrick, 2008 ▶); program(s) used to refine structure: *SHELXL97* (Sheldrick, 2008 ▶); molecular graphics: *ORTEPIII* (Burnett & Johnson, 1996 ▶); software used to prepare material for publication: *SHELXTL* (Sheldrick, 2008 ▶).

## Supplementary Material

Crystal structure: contains datablock(s) global, I. DOI: 10.1107/S1600536814011374/cv5455sup1.cif

Structure factors: contains datablock(s) I. DOI: 10.1107/S1600536814011374/cv5455Isup2.hkl

Supporting information file. DOI: 10.1107/S1600536814011374/cv5455Isup3.mol

Supporting information file. DOI: 10.1107/S1600536814011374/cv5455Isup4.cml

CCDC reference: 1003659

Additional supporting information:  crystallographic information; 3D view; checkCIF report

## Figures and Tables

**Table 1:** Hydrogen-bond geometry (Å, °)

*D*—H⋯*A*	*D*—H	H⋯*A*	*D*⋯*A*	*D*—H⋯*A*
C4—H4*A*⋯O2^i^	0.95	2.54	3.3495 (14)	143
C10—H10*A*⋯O2^ii^	0.98	2.48	3.3738 (15)	151

Symmetry codes: (i) 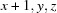; (ii) 
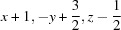
.

**Fig. 1.**
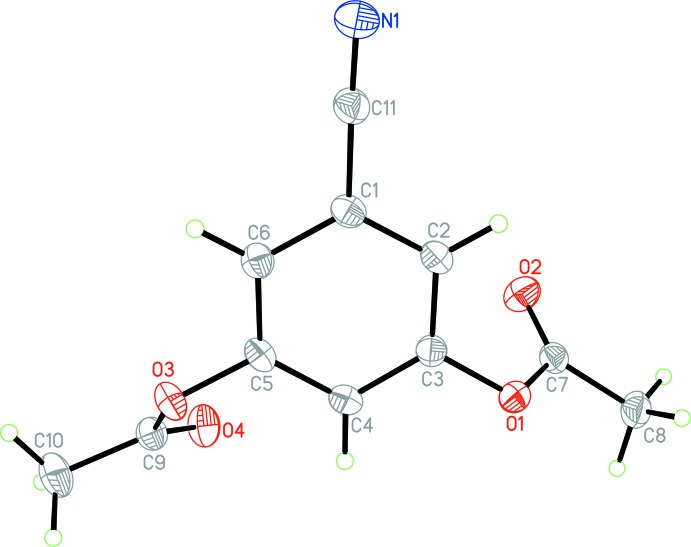
Moleculear structure of the title compound showing the atomic numbering and 50% probability displacement ellipsoids.

## supplementary crystallographic information

### 1. Comment

In the synthesis of a class of organogelators, it was necessary to shorten the synthesis of 3,5-dialkoxybenzyl amine derivatives by utilizing 5-cyano-1,3-phenylene diacetate as an intermediate. Typical synthesis of these benzyl amine derivatives started at the alkylation of methyl 3,5-dihydroxybenzoate, followed by several synthetic steps that required lithium aluminium hydride (LAH), and sodium azide (Carr, 2008). By forming the nitrile and catalytically reducing it, the hazardous chemicals (LAH, NaN_3_) are removed from the synthetic scheme creating a greener process. The 3-acetoxy-5-carbamoylphenyl acetate is dehydrated using cyaniuric acid chloride in dimethylformamide (Bhattacharyya *et al.*, 2012). The crude solid nitrile is isolated by diluting the reaction mixture with bicarbonate solution and vacuum filtration. Samples of crystaline 5-cyano-1,3-phenylene diacetate are obtained from the slow evaporation of the recytallizing solvent (acetone with 10%water).

Investigated compound (Fig. 1) crystallized in the monoclinic crystal system and the molecule occupies a general position in the unit cell. Both acetoxy groups are planar and form dihedral angles with the mean plane of the Ph-ring equal to 67.89 (4) and 53.30 (5) °, respectively and have similar geometry found in the structure of benzene-1,3,5-triyl triacetate (Haines & Hughes, 2009). In the crystal, the molecules (I) form centrosymmetric dimers through partial π-π stacking interactions between aromatic rings. Such mutual orientation of the molecules is a reason of the existance of weak intermolecular C···C contacts with distances from 3.532 Å (C1···C2) to 3.464 Å (C1···C3) that are slightly bigger than their sum of the van der Waals radii. At the same time, two weak intermolecular C—H···O hydrogen bonds with H···O distances of 2.54 and 2.48 Å (Table 1), respectively, link molecules into layers parallel to *ac* plane. The crystal packing exhibits π–π interactions between the aromatic rings proved by short intercentroid distance of 3.767 (3) Å.

### 2. Experimental

In a 250 ml round bottom flask equipped with a stir bar, 8.50 g (35.7 mmol) 3,5-diacetoxybenzamide was suspended in 25 ml of dry *N*,*N*-dimethylformamide (DMF). The reaction was placed under nitrogen. A solution of 4.40 g (23.8 mmol) 2,4,6- trichloro[1,3,5]triazine (TCT) in 15 ml of dry DMF was generated. After the TCT solution turned yellow (10 min.), it was added drop wise to the amide suspension over a period of 15 min. After 30 min. all amide dissolved. The reaction was stirred at room temperature overnight. At which time, 150 ml of 0.5 *M* sodiumbicarbonate solution was added slowly with vigorous stirring. A white solid was collected by vacuum filtration. The solid was washed with a copious amount of water and left to air dry, producing 7.9 g (97% yield) of 3-acetoxy-5-cyanophenyl acetate. m.p. 350 K (Ellis *et al.*, 1976): ^1^H NMR(300 MHz, CDCl_3_): 7.31 (d, J = 2.4 Hz, 2H). 7.20 (t, J = 2.4 Hz, 1H), 2.27 (s,CH_3_, 6H): ^13^C NMR (75 MHz CDCl_3_): 168.4, 151.4, 122.8, 120.8, 117.2, 113.8, 21.1

The nitrile was then recrystallized from the slow evaporation of acetone with 10% water, giving X-ray quality crystals.

### 3. Refinement

C-bound H atoms were placed in idealized positions (C—H = 0.95 – 0.98 Å) and allowed to ride on their parent atoms. Their positions were constrained so that the *U*_iso_(H) was equal to 1.2Ueq and 1.5 *U*_eq_ of their respective parent atoms.

### Crystal data

**Table:**

C_11_H_9_NO_4_	*F*(000) = 456
*M**_r_* = 219.19	*D*_x_ = 1.363 Mg m^−^^3^
Monoclinic, *P*2_1_/*c*	Mo *K*α radiation, λ = 0.71073 Å
Hall symbol: -P 2ybc	Cell parameters from 6321 reflections
*a* = 6.2293 (5) Å	θ = 2.8–27.1°
*b* = 21.1153 (17) Å	µ = 0.11 mm^−^^1^
*c* = 8.5989 (7) Å	*T* = 200 K
β = 109.171 (1)°	Block, colourless
*V* = 1068.32 (15) Å^3^	0.22 × 0.16 × 0.10 mm
*Z* = 4	

### Data collection

**Table:**

Bruker APEXII CCD diffractometer	2340 independent reflections
Radiation source: fine-focus sealed tube	2067 reflections with *I* > 2σ(*I*)
Graphite monochromator	*R*_int_ = 0.025
ω scans	θ_max_ = 27.1°, θ_min_ = 1.9°
Absorption correction: multi-scan (*SADABS*; Bruker, 2007)	*h* = −7→7
*T*_min_ = 0.977, *T*_max_ = 0.990	*k* = −27→27
14252 measured reflections	*l* = −10→10

### Refinement

**Table:**

Refinement on *F*^2^	Secondary atom site location: difference Fourier map
Least-squares matrix: full	Hydrogen site location: inferred from neighbouring sites
*R*[*F*^2^ > 2σ(*F*^2^)] = 0.031	H-atom parameters constrained
*wR*(*F*^2^) = 0.092	*w* = 1/[σ^2^(*F*_o_^2^) + (0.05*P*)^2^ + 0.2*P*] where *P* = (*F*_o_^2^ + 2*F*_c_^2^)/3
*S* = 1.01	(Δ/σ)_max_ = 0.001
2340 reflections	Δρ_max_ = 0.17 e Å^−^^3^
148 parameters	Δρ_min_ = −0.13 e Å^−^^3^
0 restraints	Extinction correction: *SHELXL97* (Sheldrick, 2008), Fc^*^=kFc[1+0.001xFc^2^λ^3^/sin(2θ)]^-1/4^
Primary atom site location: structure-invariant direct methods	Extinction coefficient: 0.015 (3)

### Special details

**Table:**

Geometry. All e.s.d.'s (except the e.s.d. in the dihedral angle between two l.s. planes) are estimated using the full covariance matrix. The cell e.s.d.'s are taken into account individually in the estimation of e.s.d.'s in distances, angles and torsion angles; correlations between e.s.d.'s in cell parameters are only used when they are defined by crystal symmetry. An approximate (isotropic) treatment of cell e.s.d.'s is used for estimating e.s.d.'s involving l.s. planes.
Refinement. Refinement of *F*^2^ against ALL reflections. The weighted *R*-factor *wR* and goodness of fit *S* are based on *F*^2^, conventional *R*-factors *R* are based on *F*, with *F* set to zero for negative *F*^2^. The threshold expression of *F*^2^ > σ(*F*^2^) is used only for calculating *R*-factors(gt) *etc*. and is not relevant to the choice of reflections for refinement. *R*-factors based on *F*^2^ are statistically about twice as large as those based on *F*, and *R*- factors based on ALL data will be even larger.

### Fractional atomic coordinates and isotropic or equivalent isotropic displacement parameters (Å^2^)

**Table:**

	*x*	*y*	*z*	*U*_iso_*/*U*_eq_	
O1	0.44864 (12)	0.62983 (4)	0.67935 (9)	0.0388 (2)	
C1	0.26158 (17)	0.53192 (5)	0.30796 (13)	0.0347 (2)	
N1	−0.0537 (2)	0.45024 (6)	0.17554 (15)	0.0578 (3)	
O2	0.11962 (15)	0.68031 (4)	0.55736 (11)	0.0484 (2)	
C2	0.26753 (17)	0.55886 (5)	0.45661 (13)	0.0345 (2)	
H2A	0.1601	0.5470	0.5083	0.041*	
O3	0.73998 (13)	0.60722 (4)	0.23031 (10)	0.0397 (2)	
C3	0.43337 (17)	0.60332 (5)	0.52755 (13)	0.0331 (2)	
O4	0.67580 (16)	0.71218 (4)	0.23128 (11)	0.0493 (2)	
C4	0.59239 (17)	0.62130 (5)	0.45604 (13)	0.0349 (2)	
H4A	0.7062	0.6517	0.5071	0.042*	
C5	0.58096 (17)	0.59369 (5)	0.30774 (13)	0.0339 (2)	
C6	0.41844 (18)	0.54912 (5)	0.23152 (14)	0.0355 (2)	
H6A	0.4137	0.5307	0.1297	0.043*	
C7	0.27412 (18)	0.66893 (5)	0.67974 (13)	0.0361 (2)	
C8	0.3085 (2)	0.69393 (6)	0.84782 (15)	0.0490 (3)	
H8A	0.1718	0.7167	0.8487	0.073*	
H8B	0.4387	0.7229	0.8795	0.073*	
H8C	0.3377	0.6587	0.9261	0.073*	
C9	0.77434 (19)	0.66931 (5)	0.19634 (14)	0.0374 (3)	
C10	0.9438 (2)	0.67242 (6)	0.10879 (19)	0.0531 (3)	
H10A	0.9422	0.7149	0.0623	0.080*	
H10B	0.9055	0.6410	0.0199	0.080*	
H10C	1.0956	0.6634	0.1864	0.080*	
C11	0.0867 (2)	0.48624 (5)	0.23206 (15)	0.0410 (3)	

### Atomic displacement parameters (Å^2^)

**Table:**

	*U*^11^	*U*^22^	*U*^33^	*U*^12^	*U*^13^	*U*^23^
O1	0.0349 (4)	0.0484 (5)	0.0320 (4)	0.0001 (3)	0.0095 (3)	−0.0013 (3)
C1	0.0325 (5)	0.0302 (5)	0.0416 (6)	−0.0003 (4)	0.0125 (4)	0.0017 (4)
N1	0.0610 (7)	0.0529 (6)	0.0611 (7)	−0.0218 (5)	0.0224 (6)	−0.0100 (5)
O2	0.0467 (5)	0.0513 (5)	0.0431 (5)	0.0103 (4)	0.0091 (4)	−0.0065 (4)
C2	0.0307 (5)	0.0358 (5)	0.0392 (6)	0.0002 (4)	0.0142 (4)	0.0042 (4)
O3	0.0395 (4)	0.0349 (4)	0.0539 (5)	−0.0003 (3)	0.0278 (4)	0.0008 (3)
C3	0.0309 (5)	0.0361 (5)	0.0322 (5)	0.0034 (4)	0.0101 (4)	0.0021 (4)
O4	0.0627 (6)	0.0390 (4)	0.0547 (5)	0.0099 (4)	0.0309 (4)	0.0054 (4)
C4	0.0280 (5)	0.0349 (5)	0.0406 (6)	−0.0003 (4)	0.0099 (4)	0.0017 (4)
C5	0.0303 (5)	0.0328 (5)	0.0425 (6)	0.0028 (4)	0.0171 (4)	0.0044 (4)
C6	0.0379 (5)	0.0320 (5)	0.0392 (5)	0.0024 (4)	0.0160 (4)	−0.0002 (4)
C7	0.0380 (5)	0.0363 (5)	0.0370 (6)	−0.0058 (4)	0.0163 (4)	−0.0014 (4)
C8	0.0608 (8)	0.0515 (7)	0.0400 (6)	−0.0111 (6)	0.0238 (6)	−0.0082 (5)
C9	0.0389 (6)	0.0358 (5)	0.0394 (6)	−0.0003 (4)	0.0153 (4)	0.0021 (4)
C10	0.0602 (8)	0.0441 (7)	0.0695 (9)	−0.0025 (6)	0.0411 (7)	0.0047 (6)
C11	0.0437 (6)	0.0377 (6)	0.0443 (6)	−0.0053 (5)	0.0183 (5)	−0.0017 (5)

### Geometric parameters (Å, º)

**Table:**

O1—C7	1.3661 (13)	C4—C5	1.3826 (15)
O1—C3	1.3944 (12)	C4—H4A	0.9500
C1—C2	1.3884 (15)	C5—C6	1.3800 (15)
C1—C6	1.3930 (15)	C6—H6A	0.9500
C1—C11	1.4409 (15)	C7—C8	1.4866 (16)
N1—C11	1.1402 (15)	C8—H8A	0.9800
O2—C7	1.1943 (14)	C8—H8B	0.9800
C2—C3	1.3809 (15)	C8—H8C	0.9800
C2—H2A	0.9500	C9—C10	1.4859 (16)
O3—C9	1.3752 (13)	C10—H10A	0.9800
O3—C5	1.3926 (12)	C10—H10B	0.9800
C3—C4	1.3797 (14)	C10—H10C	0.9800
O4—C9	1.1865 (13)		
			
C7—O1—C3	115.80 (8)	O2—C7—O1	122.08 (10)
C2—C1—C6	121.12 (10)	O2—C7—C8	127.04 (11)
C2—C1—C11	118.61 (9)	O1—C7—C8	110.88 (10)
C6—C1—C11	120.26 (10)	C7—C8—H8A	109.5
C3—C2—C1	118.37 (9)	C7—C8—H8B	109.5
C3—C2—H2A	120.8	H8A—C8—H8B	109.5
C1—C2—H2A	120.8	C7—C8—H8C	109.5
C9—O3—C5	118.81 (8)	H8A—C8—H8C	109.5
C4—C3—C2	122.18 (10)	H8B—C8—H8C	109.5
C4—C3—O1	117.94 (9)	O4—C9—O3	122.96 (10)
C2—C3—O1	119.84 (9)	O4—C9—C10	127.40 (11)
C3—C4—C5	117.91 (10)	O3—C9—C10	109.62 (9)
C3—C4—H4A	121.0	C9—C10—H10A	109.5
C5—C4—H4A	121.0	C9—C10—H10B	109.5
C6—C5—C4	122.22 (9)	H10A—C10—H10B	109.5
C6—C5—O3	116.03 (9)	C9—C10—H10C	109.5
C4—C5—O3	121.68 (9)	H10A—C10—H10C	109.5
C5—C6—C1	118.20 (10)	H10B—C10—H10C	109.5
C5—C6—H6A	120.9	N1—C11—C1	178.16 (13)
C1—C6—H6A	120.9		

### Hydrogen-bond geometry (Å, º)

**Table:**

*D*—H···*A*	*D*—H	H···*A*	*D*···*A*	*D*—H···*A*
C4—H4*A*···O2^i^	0.95	2.54	3.3495 (14)	143
C10—H10*A*···O2^ii^	0.98	2.48	3.3738 (15)	151

Symmetry codes: (i) *x*+1, *y*, *z*; (ii) *x*+1, −*y*+3/2, *z*−1/2.
